# Metabolic Profiling of Epigenetic Aging and Its Associations With Aging‐Related Phenotypes and Modifiable Lifestyle Factors

**DOI:** 10.1111/acel.70657

**Published:** 2026-08-23

**Authors:** Xunying Zhao, Tianpei Ma, Maoyao Xia, Xinyang Dui, Yangdan Zhong, Xin Chen, Yang Qu, Bowen Lei, Qingwen Zhao, Xueyao Wu, Haiyu Yan, Xingyu Zhang, Ke Jiang, Lin Hu, Lu Long, Mengyu Fan, Jiaqiang Liao, Tao Zhang, Xia Jiang, Jiayuan Li, Ben Zhang

**Affiliations:** ^1^ Department of Epidemiology and Health Statistics and West China Institute of Preventive and Medical Integration for Major Diseases, West China School of Public Health and West China Fourth Hospital Sichuan University Chengdu Sichuan China; ^2^ Department of Nutrition and Food Hygiene, West China School of Public Health and West China Fourth Hospital Sichuan University Chengdu China; ^3^ West China School of Public Health and West China Fourth Hospital Sichuan University Chengdu China

**Keywords:** aging‐related phenotypes, biological aging, epigenetic aging, metabolomics, modifiable factors

## Abstract

Epigenetic aging biomarkers are well‐established hallmarks of biological aging, yet their metabolic underpinnings remain largely unexplored. Here, we characterized metabolic signatures associated with five epigenetic aging biomarkers (HorvathAge, HannumAge, DNAmPhenoAge, DunedinPACE, and DNAmTL) and examined their clinical relevance and potential determinants in 7162 Chinese older adults from two cohorts (primary and validation). We observed both shared and distinct metabolic associations across epigenetic aging biomarkers. Metabolic signatures of epigenetic aging biomarkers were derived using elastic net regression, showing moderate correlations with the corresponding epigenetic aging biomarkers (*r* = 0.21–0.36 in internal testing set, *p* < 0.05), with external replication further validating metabolic signatures of DNAmPhenoAge, DunedinPACE, and DNAmTL (*r* = 0.18–0.29, *p* < 0.05). These five metabolic signatures of epigenetic age acceleration (EAA) exhibited 279 significant associations with aging‐related phenotypes including higher disease risk, poorer health status, and adverse clinical indicators. Gallstones, chronic kidney disease, and hepatitis, along with renal‐, hepatic‐ and metabolic‐related clinical indicators, were consistently associated with multiple metabolic signatures of EAA. Smoking status, alcohol consumption, body mass index (BMI), and physical activity were identified as modifiable lifestyle factors associated with metabolic signatures of EAA, with BMI showing the most consistent associations. Metabolic signatures of DunedinPACE and DNAmPhenoAA exhibited the most extensive associations with aging‐related phenotypes and modifiable lifestyle factors in both primary and validation cohorts. These findings provide novel insights into the metabolic correlates of epigenetic aging biomarkers and underscore the potential of metabolomics‐informed metrics of epigenetic aging as informative indicators of physiological decline and lifestyle effects.

## Graphical abstract

This study provides novel insights into the metabolic correlates of epigenetic aging biomarkers and underscores the potential of metabolomics‐informed metrics of epigenetic aging as informative indicators of physiological decline and lifestyle effects.

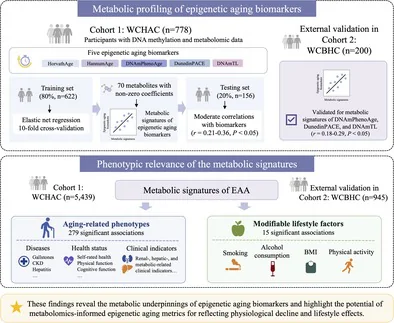

## 1 Introduction

Aging is a complex biological process characterized by the gradual accumulation of molecular and cellular damage, leading to functional decline and increased risks of diseases (Guo et al. 2022). The heterogeneity in how individuals age has prompted the development of indicators that reflect biological age rather than merely chronological age. Among these, epigenetic aging biomarkers, which integrate methylation data across cytosine‐phosphate‐guanine (CpG) sites to predict chronological age and/or age‐related endpoints, have emerged as promising tools (Duan et al. 2022). Multiple epigenetic aging biomarkers have been developed, including first‐generation epigenetic clocks predicting chronological age (e.g., HorvathAge and HannumAge) (Hannum et al. 2013; Horvath 2013), second‐generation epigenetic clocks predicting morbidity and mortality (e.g., DNA methylation‐based PhenoAge, [DNAmPhenoAge]) (Levine et al. 2018), and third‐generation epigenetic clocks estimating the pace of aging (e.g., DunedinPACE) (Belsky et al. 2022). In addition, DNA methylation‐based telomere length (DNAmTL) has been developed to proxy leukocyte telomere length, providing additional epigenetic insights into cellular aging and replicative capacity (Lu et al. 2019; Liang et al. 2024). Despite their differing emphases, these complementary epigenetic aging biomarkers have been widely linked to adverse health outcomes and collectively provide a multidimensional perspective on biological aging (Duan et al. 2022; Moqri et al. 2023).

Although epigenetic aging biomarkers capture DNA methylation patterns linked to biological aging, the broader biological and physiological processes associated with these biomarkers and their relevance to functional aspects of aging remain incompletely understood (Mavromatis et al. 2023; Bell et al. 2019; Field et al. 2018). Metabolomics, which quantifies the small‐molecule metabolites representing the products of complex regulatory networks, offers a valuable analytical tool (Jacob et al. 2019). By sensitively reflecting subtle changes in gene expression, protein translation, and environmental exposures, the metabolome can illuminate biological processes associated with epigenetic aging biomarkers and provide a functional snapshot of the physiological state (Field et al. 2018; Qiu et al. 2023; Krumsiek et al. 2016; Suhre and Zaghlool 2021). Thus, identifying metabolomic profiles associated with epigenetic aging biomarkers may enhance the biological interpretability of these biomarkers. Moreover, due to the dynamic and potentially modifiable nature, metabolites may provide more responsive and accessible molecular readouts of short‐term physiological changes (Qiu et al. 2023; Lin et al. 2024). Although several metabolites—particularly lipids, amino acids, and intermediates of energy metabolism—have been implicated in aging (Sebastiani et al. 2024; Liu et al. 2023; Bartke et al. 2021), relatively few studies have explored the metabolomic correlates of epigenetic aging biomarkers. Furthermore, while it is widely acknowledged that different epigenetic aging biomarkers may capture distinct aspects of biological aging (Duan et al. 2022; Moqri et al. 2023; Kuiper et al. 2023; Thomas et al. 2024), the specific metabolic mechanisms underlying each remain largely unelucidated.

In this study, we used DNA methylation, metabolomics, and phenotypic data from the West China Health and Aging Cohort (WCHAC) study to investigate metabolic profiles of epigenetic aging biomarkers, along with the clinical relevance and potential determinants of the metabolic profiles. We first validated the epigenetic aging biomarkers in Chinese older adults and identified individual plasma metabolites and integrative metabolic signatures associated with epigenetic aging biomarkers to enhance the understanding of the metabolic mechanisms linked to epigenetic aging. We then examined associations of these metabolic signatures with aging‐related phenotypes and modifiable lifestyle factors to assess their phenotypic relevance. External validation was conducted using data from the West China Bone Health Cohort (WCBHC) study.

## 2 Methods

### 2.1 Study Design and Population

**FIGURE 1:**
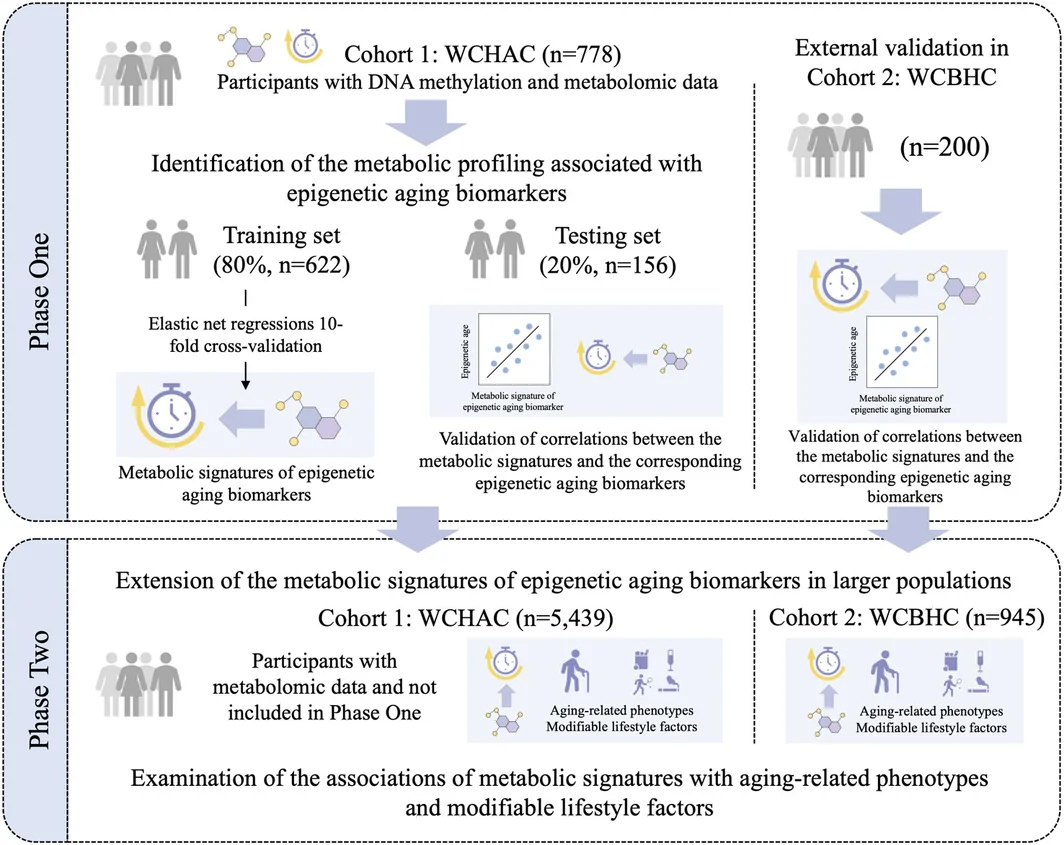
The design of the study. Primary analyses were conducted in WCHAC in two phases: Phase One identified metabolic signatures associated with epigenetic aging biomarkers using 778 participants with both methylation and metabolomic data; Phase Two evaluated clinical relevance and determinants of these signatures in 5439 participants with metabolomic data only. External validation was conducted in WCBHC. WCBHC, West China Bone Health Cohort; WCHAC, West China Health and Aging Cohort.

Primary analyses were conducted using baseline data from the WCHAC, which was initiated in 2022 and enrolled 10,626 community‐dwelling adults aged ≥ 54 years from Pidu District, Chengdu (Zhao et al. 2025; Wu et al. 2025). Data collection included questionnaire surveys, physical measurements, and fasting blood samples in EDTA tubes. The analyses were conducted in two phases among participants with available omics data (Figure 1). Phase One included 778 participants with both methylation and metabolomic data (after excluding 6 participants with sex discrepancy and 16 with cancer) to identify metabolic signatures associated with epigenetic aging biomarkers. Phase Two evaluated the clinical relevance and potential determinants of the integrative metabolic signatures in a larger, non‐overlapping sample of 5439 participants with metabolomic data only (after excluding 252 with cancer or missing responses). Details of the inclusion and exclusion criteria are described in Figure S1. The study followed the STROBE (Strengthening the Reporting of Observational Studies in Epidemiology) guidelines (Appendix S1, https://www.strobe‐statement.org/).

Replication analyses were conducted using baseline data from the WCBHC, an ongoing study initiated in 2023. As of December 2024, the WCBHC had enrolled 1388 individuals aged ≥ 25 years with normal or reduced bone mass from West China Fourth Hospital, Chengdu. After excluding those with cancer or aged < 50 years, 200 participants with both methylation and metabolomic data were used to validate metabolite–epigenetic aging associations, and 945 participants with metabolomic data were used to validate metabolic signature–phenotype associations (Figure S1).

### 2.2 Measurement of DNA Methylation and Calculation of Epigenetic Aging Biomarkers

Genomic DNA was extracted from peripheral blood samples using the MagPure Blood DNA KF Kit (Magen, China). DNA methylation profiling was conducted using the Infinium Methylation Screening Array (Illumina, CA, USA), an Illumina commercial array covering ~270,000 loci across the genome (Goldberg et al. 2025). Briefly, after quality control procedures (detailed in Appendix S2), a total of 778 participants and 236,685 CpG probes remained. After normalization and batch correction, the *β*‐values were used as input for calculating epigenetic aging biomarkers.

We included five established epigenetic aging biomarkers, comprising four epigenetic clocks (HorvathAge (Horvath 2013), HannumAge (Hannum et al. 2013), DNAmPhenoAge (Levine et al. 2018), and DunedinPACE (Belsky et al. 2022)) and one DNAm estimator of telomere length (DNAmTL) (Lu et al. 2019), with high CpG site matching rates of 90.71%–99.42% in our data relative to the original algorithms (Table S1). The biomarkers were calculated using the methylclock R package (Pelegí‐Sisó et al. 2021) and the DunedinPACE R package (Belsky et al. 2022). For all epigenetic aging biomarkers except DunedinPACE, we calculated epigenetic age acceleration (EAA) as the residuals from regressing each biomarker on chronological age (Noroozi et al. 2021). Thus, EAA represents the deviation of each biomarker from that expected for chronological age. Positive values of HorvathAA, HannumAA, and DNAmPhenoAA indicate accelerated epigenetic aging; for DNAmTL adjusted by age (DNAmTLadjAge), the interpretation is reversed, as a negative value indicates shorter‐than‐expected telomere length and accelerated biological aging. DunedinPACE was not residualized because it inherently represents the pace of aging itself, with higher DunedinPACE indicating a faster pace of aging.

### 2.3 Measurement of Plasma Metabolites

Targeted quantitative metabolomics was conducted to quantify the absolute concentrations of small‐molecule metabolites in plasma samples using ultra performance liquid chromatography–tandem mass spectrometry (UPLC‐MS/MS; TQ‐S, Waters, USA) (Wu et al. 2026). A total of 221 common metabolites were quantified based on their relevance to human metabolic health and their detection rates in blood samples (Yang et al. 2025; Castro et al. 2022; Franceschi et al. 2018), covering amino acids, benzenoids and aromatic compounds, bile acids, carbohydrates, carnitines, fatty acids, indoles, imidazoles, organic acids, pyridines, furans, and steroids (Table S2). Prior to analysis, metabolite concentrations were log‐transformed to reduce skewed distributions and then standardized to *z*‐scores with a mean of 0 and a standard deviation (SD) of 1. Detailed procedures for metabolomic measurements are provided in Appendix S2.

### 2.4 Assessment of Aging‐Related Phenotypes

We included a broad spectrum of aging‐related phenotypes, encompassing self‐reported diseases, objective and subjective health status, and clinical indicators reflecting multiple organ systems and physiological functions (Table S3). Self‐reported diseases included common chronic conditions such as metabolic, cardiovascular, respiratory, digestive, genitourinary, and musculoskeletal diseases. Objective and subjective health status assessments included measures of functional limitations, cognitive impairment, frailty, hearing impairment, anxiety and depression symptoms, grip strength, walking speed, and self‐rated health scores. Clinical indicators encompassed commonly used hematological and physical examination measures from multiple physiological systems, including immune‐, metabolic‐, renal‐, hepatic‐, cardiovascular‐, endocrine‐, and body composition‐related indicators. All clinical indicators were log‐transformed. Variables with missing data exceeding 20% were excluded from analyses, whereas variables with less than 20% missing data were imputed using multivariate chained equations (Van Buuren and Groothuis‐Oudshoorn 2011). Detailed methodologies and definitions of all aging‐related phenotypes are provided in Appendix S2.

### 2.5 Modifiable Lifestyle Factors and Covariates

Trained researchers conducted face‐to‐face questionnaire surveys to collect socio‐demographic information (chronological age, sex, educational attainment, and major occupation), modifiable lifestyle factors (smoking status, alcohol consumption, body mass index [BMI], physical activity, sleep quality, and dietary habits) (Huang, da, et al. 2025; Chen et al. 2023), and medication use history (regular use of diabetes and cardiovascular disease drugs in the past 6 months). Details on the definitions of covariates and modifiable lifestyle factors are provided in Appendix S2.

### 2.6 Statistical Analyses

#### 2.6.1 Validation of Epigenetic Aging Biomarkers in Chinese Older Adults

Participant characteristics were presented as mean ± SD or median (interquartile range [IQR]) for continuous variables, and as frequencies and percentages for categorical variables. Spearman correlation coefficients were calculated to assess the relationship between chronological age and each epigenetic aging biomarker. We evaluated the associations between each EAA and aging‐related phenotypes using logistic regression models for dichotomous outcomes and linear regression models for continuous outcomes. All models were adjusted for chronological age, sex, educational attainment, occupation, smoking status, alcohol consumption, BMI, physical activity, sleep quality, and dietary habits. Subsequently, we examined the associations between modifiable factors and each EAA using linear regression models, adjusting for chronological age, sex, educational attainment, occupation, and all modifiable lifestyle factors simultaneously. Benjamini‐Hochberg false discovery rate (FDR) correction was applied separately for aging‐related phenotypes and modifiable lifestyle factors within each EAA, with *p* < 0.05 considered nominally significant and *p*_FDR_ < 0.05 considered statistically significant (Benjamini and Hochberg 1995).

#### 2.6.2 Metabolome‐Wide Associations of Epigenetic Aging Biomarkers

Spearman correlation analysis was first used to describe correlation patterns between metabolites and each epigenetic aging biomarker. Linear regression models were used to estimate associations between individual plasma metabolites (per 1‐SD increase) and each epigenetic aging biomarker, adjusting for the aforementioned covariates and additionally for medication use for diabetes and cardiovascular diseases due to the potential metabolic effects of these treatments. FDR correction was applied within each epigenetic aging biomarker, with *p* < 0.05 considered nominally significant and *p*_FDR_ < 0.05 considered statistically significant (Benjamini and Hochberg 1995). Metabolites with nominal significance (*p* < 0.05) were considered candidate metabolites for each epigenetic aging biomarker in subsequent analyses. Enrichment analysis was performed using MetaboAnalyst 6.0 and the Kyoto Encyclopedia of Genes and Genomes (KEGG) database to identify metabolic pathways enriched among candidate metabolites for each epigenetic aging biomarker (Pang et al. 2024).

#### 2.6.3 Identification of Metabolic Signatures for Epigenetic Aging Biomarkers

We identified metabolic signatures for epigenetic aging biomarkers using elastic net penalized linear regression based on the corresponding candidate metabolites. Elastic net regression combines Lasso and Ridge penalties to reduce model overfitting (Zou and Hastie 2005). We set alpha to 0.5, equally weighting the L1 and L2 penalties. Participants were randomly divided into training and testing sets with an 8:2 split. The penalty parameter lambda was selected using 10‐fold cross‐validation in the training set, choosing the lambda value that minimized the mean squared error. For each epigenetic aging biomarker, the metabolic signature was calculated as the weighted sum of selected metabolites, using the non‐zero coefficients from the elastic net model as weights, and was then standardized to a *z*‐score. Spearman correlation coefficients were used to examine correlations between each metabolic signature and the corresponding epigenetic aging biomarker in both the training and testing sets. For all epigenetic aging biomarkers except DunedinPACE, we derived the corresponding metabolic signatures of EAA (defined as age‐adjusted metabolic signatures) as the residuals from regressing each metabolic signature of epigenetic aging biomarker on chronological age. Metabolic signatures of EAA were standardized to *z*‐scores.

#### 2.6.4 Clinical Relevance and Lifestyle Determinants of Metabolic Signatures

After deriving metabolic signatures in 778 participants (referred to as Phase One), we evaluated their clinical relevance and potential lifestyle determinants in a larger, non‐overlapping sample (referred to as Phase Two). Associations between metabolic signatures of EAA and aging‐related phenotypes were assessed using linear or logistic regression models, depending on the outcome type. Associations between modifiable lifestyle factors and metabolic signatures of EAA were examined using linear regression models. All models were adjusted for the same covariates described above, with additional adjustment for medication use. Multiple testing was controlled using FDR correction separately for aging‐related phenotypes and modifiable lifestyle factors within each metabolic signature of EAA (Benjamini and Hochberg 1995). An association with *p* < 0.05 was considered nominally significant, and *p*_FDR_ < 0.05 was considered statistically significant. To evaluate the robustness of the results, we conducted several sensitivity analyses: (i) stratified analyses by sex (female and male) and age group (< 70 and ≥ 70 years); and (ii) repeated the analyses among Phase One participants and additionally conducted pooled analyses combining Phase One and Phase Two participants.

#### 2.6.5 Replication Analyses

We conducted replication analyses in an independent cohort (WCBHC) to validate the metabolic signatures derived from the WCHAC data and their associations with phenotypes. Methylation and metabolomics measurements, as well as definitions of covariates and modifiable lifestyle factors, were consistent with those used in WCHAC (Appendix S2). Aging‐related phenotypes were defined in the same manner as in WCHAC; however, some phenotypes were not included due to data unavailability or missing rates exceeding 20% (Appendix S2 and Table S3). All analyses applied the same modeling framework and covariate adjustment strategy as the primary analyses, with the replication analyses in WCBHC additionally controlling for fasting status because WCBHC collected random blood samples. Robustness was assessed based on the consistency of association directions and statistical significance. As a sensitivity analysis, we also repeated the analyses including WCBHC participants younger than 50 years, resulting in a total of 1005 participants.

## 3 Results

### 3.1 Validation of Epigenetic Aging Biomarkers in Chinese Older Adults

**FIGURE 2:**
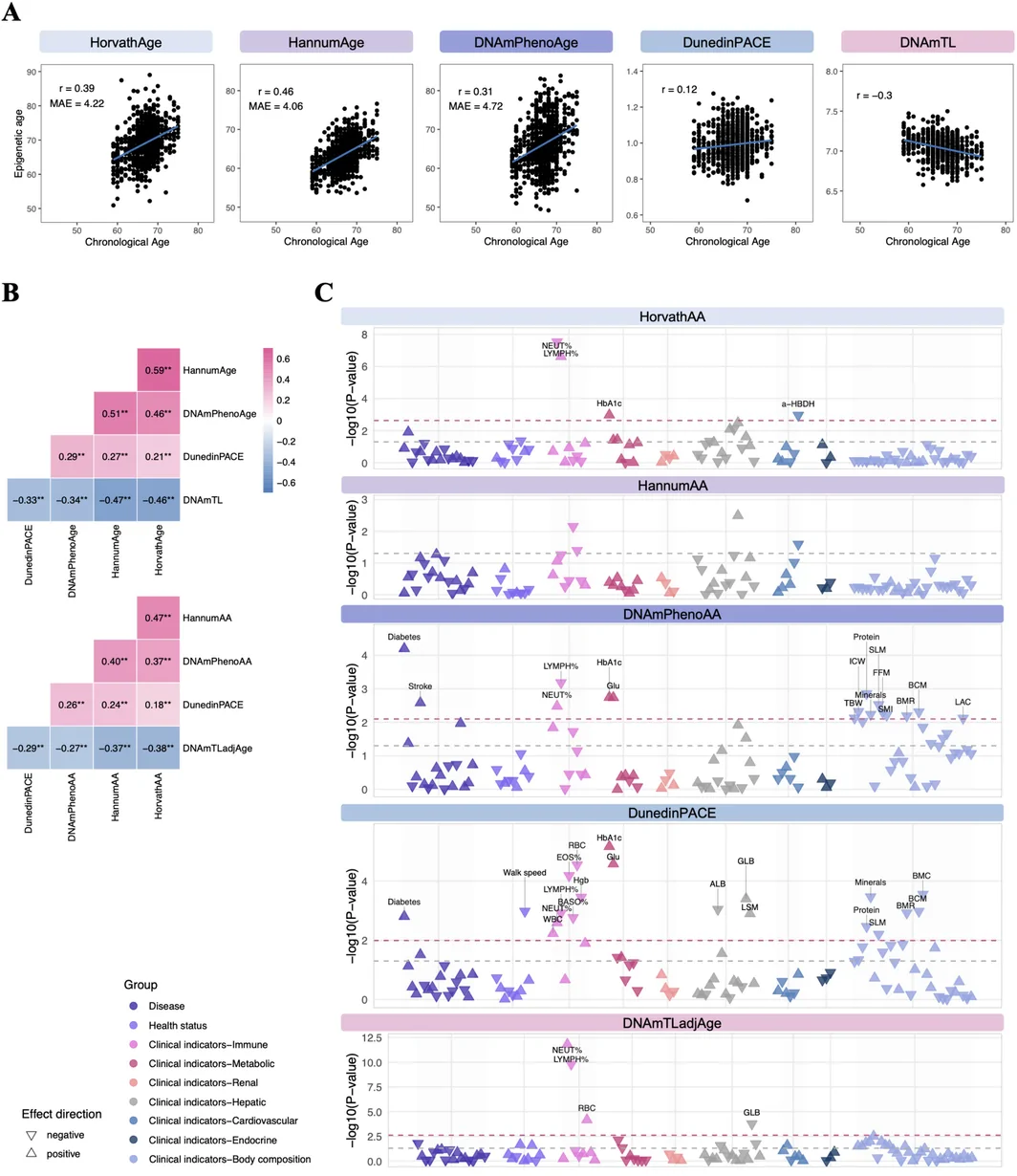
Validation of epigenetic aging biomarkers in Chinese older adults. (A) Scatter plot showing the relationship between epigenetic aging biomarkers and chronological age. Spearman correlation coefficients are indicated. Mean absolute errors (MAE) are shown for HorvathAge, HannumAge, and DNAmPhenoAge. (B) Heatmap of inter‐relationships between epigenetic aging biomarkers. Pink represents positive correlations, and blue represents negative correlations (Spearman correlation coefficients). Asterisks (**) indicate *p*_FDR_ < 0.05. (C) Manhattan plot showing associations between epigenetic age acceleration and aging‐related phenotypes across multiple physiological systems. Upper triangles represent positive effect sizes and lower triangles represent negative effect sizes. The gray dashed line indicates *p* of 0.05 and the red dashed line indicates *p*_FDR_ of 0.05. Phenotypes with *p*_FDR_ < 0.05 are labeled with their names in the plot. Color coding for aging‐related phenotypes and effect direction are shown in the figure legend.

Among 778 participants in Phase One (49.87% female, median age: 67 years), the median (IQR) values were 68.76 (65.65–72.42) for HorvathAge, 63.36 (60.96–66.25) for HannumAge, 66.07 (62.01–70.18) for DNAmPhenoAge, 0.99 (0.93–1.05) for DunedinPACE, and 7.05 (6.95–7.14) for DNAmTL (Table S4). All epigenetic aging biomarkers were significantly positively correlated with chronological age, except for DNAmTL, which exhibited an inverse correlation as expected (|*r*| = 0.12–0.46, all *p*_FDR_ < 0.05, Figure 2A). Significant inter‐correlations were also observed among the epigenetic aging biomarkers (|*r*| = 0.21–0.59) and among the EAA (|*r*| = 0.18–0.47, all *p*_FDR_ < 0.05, Figure 2B).

A total of 44 significant associations were identified between EAA and aging‐related phenotypes after FDR correction, with distinct patterns observed across different EAA (Figure 2C and Table S5). DunedinPACE exhibited the most extensive associations (*n* = 20), including diabetes, slow walking speed, and clinical indicators spanning immune‐, metabolic‐, hepatic‐, and body composition‐related domains. DNAmPhenoAA showed 16 significant associations, involving diabetes, stroke, as well as immune‐, metabolic‐, and body composition‐related indicators. Both HorvathAA and DNAmTLadjAge were each associated with four indicators, primarily within the immune‐related domain. No significant associations were observed for HannumAA after multiple testing correction. As for modifiable lifestyle factors, we observed that current smoking and overweight/obesity were significantly associated with faster DunedinPACE after FDR correction (*p*_FDR_ < 0.05; Table S6).

### 3.2 Metabolome‐Wide Associations of Epigenetic Aging Biomarkers

**FIGURE 3:**
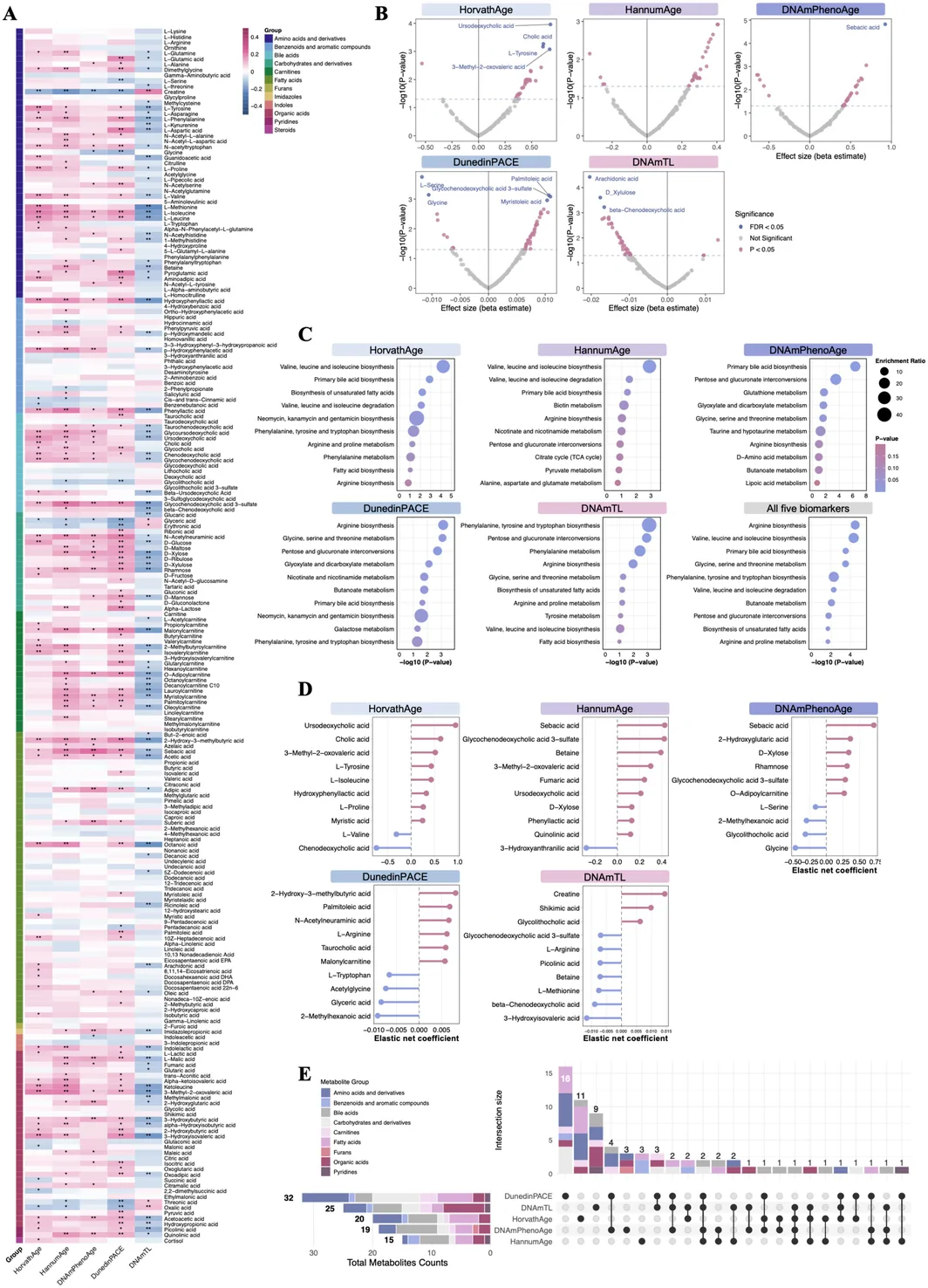
Associations between epigenetic aging biomarkers and plasma metabolites. (A) Heatmap of Spearman correlations between metabolites and each epigenetic aging biomarker. Pink represents positive correlations and blue represents negative correlations. One asterisk (*) indicates *p* < 0.05 and two asterisks (**) indicate *p*_FDR_ < 0.05. (B) Volcano plot of associations between metabolites and each epigenetic aging biomarker. Pink dots represent *p* < 0.05 and blue dots represent *p*_FDR_ < 0.05. (C) Bubble plot of metabolite set enrichment analysis showing the top 10 enriched pathways based on candidate metabolites. (D) Lollipop plot showing the top 10 metabolites with non‐zero coefficients and their weights in the elastic net regression model for each metabolic signature. Pink represents positive weights and blue represents negative weights. (E) Upset plot of intersections between metabolites with non‐zero coefficients across five metabolic signatures of epigenetic aging biomarkers.

Spearman correlation analysis revealed that metabolites correlated with epigenetic aging biomarkers were predominantly from the categories of amino acids, bile acids, carbohydrates, carnitines, fatty acids, and organic acids and their derivatives (Figure 3A and Table S7). After adjusting for potential confounders, 92 metabolites were nominally associated (*p* < 0.05) with at least one epigenetic aging biomarker and were considered corresponding candidate metabolites. Specifically, 43 candidate metabolites were identified for DunedinPACE, 41 for DNAmTL, 27 for HannumAge, and 26 each for DNAmPhenoAge and HorvathAge. Among these, 13 remained statistically significant after FDR correction (Figure 3B and Table S8). DunedinPACE was significantly associated with five metabolites, including L‐serine, glycine, myristoleic acid, palmitoleic acid, and glycochenodeoxycholic acid 3‐sulfate. HorvathAge followed with four metabolites (L‐tyrosine, 3‐methyl‐2‐oxovaleric acid, ursodeoxycholic acid, and cholic acid), DNAmTL with three (D‐xylulose, arachidonic acid, and beta‐chenodeoxycholic acid), and DNAmPhenoAge with one (sebacic acid).

Metabolite set enrichment analysis based on candidate metabolites for each epigenetic aging biomarker identified enriched pathways mainly related to energy metabolism, antioxidation, inflammation, nerve function, and immune regulation, including three pathways for DunedinPACE, two each for DNAmPhenoAge and DNAmTL, and one for HorvathAge (Figure 3C and Table S9). Notably, pentose and glucuronate interconversions emerged as a common enriched pathway for DNAmPhenoAge, DunedinPACE, and DNAmTL, suggesting a potential role of altered carbohydrate metabolism and detoxification processes in biological aging. When considering all candidate metabolites across the five epigenetic aging biomarkers, four pathways remained significant after FDR correction: arginine biosynthesis, valine, leucine and isoleucine biosynthesis, primary bile acid biosynthesis, and glycine, serine and threonine metabolism. These pathways are implicated in maintaining nitrogen balance, regulating oxidative stress, modulating mitochondrial function, and preserving metabolic homeostasis, all of which are critical in aging‐related biological dysfunction.

### 3.3 The Metabolic Profiling of Epigenetic Aging Biomarkers

Elastic net regression selected 70 metabolites with non‐zero coefficients for at least one epigenetic aging biomarker, including 20 for HorvathAge, 15 for HannumAge, 19 for DNAmPhenoAge, 32 for DunedinPACE, and 25 for DNAmTL (Figure 3D,E and Table S10). D‐xylose, glycolithocholic acid, and ursodeoxycholic acid were each selected for four epigenetic aging biomarkers, with coefficient directions consistently indicating accelerated or decelerated biological aging. These shared metabolites highlighted overlapping signals related to carbohydrate and bile acid metabolism. Despite some overlaps, each epigenetic aging biomarker also exhibited distinct metabolic features: HorvathAge with branched‐chain amino acids (BCAA) (e.g., L‐valine, L‐isoleucine); HannumAge with aromatic compound signatures (e.g., phenyllactic acid, 3‐hydroxyanthranilic acid); DNAmPhenoAge with specific metabolites including the amino acid ornithine and furan compounds; DunedinPACE with the broadest metabolic coverage, reflecting diverse fatty acids, carbohydrate derivatives, and amino acids including neurotransmitter precursors (e.g., L‐tryptophan); and DNAmTL with metabolites related to cellular energy metabolism, such as creatine.

The metabolic signature for each epigenetic aging biomarker was significantly correlated with the corresponding biomarker in both the training (*r* = 0.33–0.45, *p* < 0.05) and testing sets (*r* = 0.21–0.36, *p* < 0.05, Table S11). Metabolic signatures explained 11.1%–19.8% of the variance (*R* ^2^) in the corresponding epigenetic aging biomarkers in the training set and 4.4%–13.0% in the testing set. The absolute inter‐correlation between metabolic signatures of epigenetic aging biomarkers ranged from 0.34 to 0.71 in the training set and 0.34–0.69 in the testing set (Figure S2). We also examined the concordance between the metabolite coefficient directions in metabolic signatures of epigenetic aging biomarkers and those in four previously established metabolic age clocks (MetaboAge2016 (Van Den Akker et al. 2020), MetaboAge2020 (Bizzarri et al. 2023), MetaboHealth (Deelen et al. 2019), and MetaboAgeMort (Jia et al. 2024)). Seven metabolites were also reported in the established metabolic aging clocks. Five of these metabolites had coefficient directions in the metabolic signatures that matched those in all previous clocks, whereas two matched some of the previous clocks, supporting the plausibility of the metabolic signatures (Table S10).

### 3.4 Associations With Aging‐Related Phenotypes and Modifiable Factors

**FIGURE 4:**
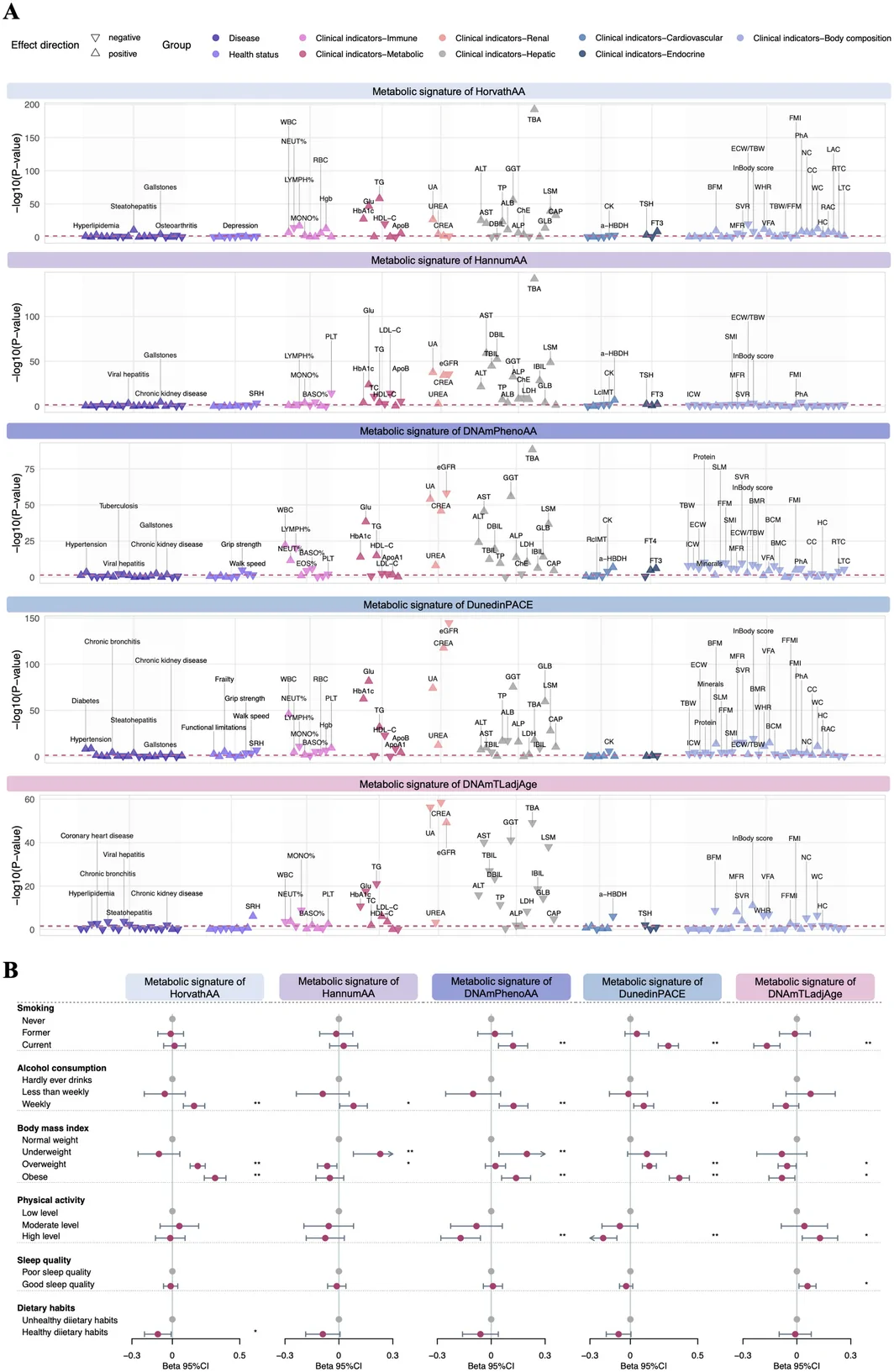
Associations of metabolic signatures of EAA with aging‐related phenotypes and modifiable lifestyle factors. (A) Manhattan plot showing associations between metabolic signatures of EAA and aging‐related phenotypes across multiple physiological systems. Upper triangles represent positive effect sizes and lower triangles represent negative effect sizes. The gray dashed line indicates *p* of 0.05 and the red dashed line indicates *p*_FDR_ of 0.05. Phenotypes with *p*_FDR_ < 0.05 are labeled with their names in the plot. Color coding for aging‐related phenotypes and effect direction are shown in the figure legend. (B) Forest plot of associations between modifiable risk factors and each metabolic signature of EAA. Circles represent the point estimates of beta, and error bars represent 95% confidence intervals. Gray represents the reference groups, whereas dark pink represents estimates of each group compared to the reference group. Two asterisks (**) represent *p*_FDR_ < 0.05, and one asterisk (*) represents *p* < 0.05. EAA, epigenetic age acceleration.

A total of 5439 participants (55.47% female, median age: 68 years) were included in Phase Two (Table S12). Metabolic signatures of EAA exhibited significant associations across all aging‐related phenotype categories, with 279 associations remaining statistically significant after FDR correction (out of 302 nominally significant associations) (Figure 4A and Table S13). Metabolic signature of DunedinPACE showed the largest number of significant associations (*n* = 68), followed by metabolic signature of DNAmPhenoAA (*n* = 64), metabolic signature of HorvathAA (*n* = 53), metabolic signature of DNAmTLadjAge (*n* = 48), and metabolic signature of HannumAA (*n* = 46). These associations mostly aligned with biological expectations, with higher values of metabolic signatures of EAA generally associated with adverse health outcomes (increased disease risk, poorer health status, and unfavorable clinical indicators), except for metabolic signature of DNAmTLadjAge which showed inverse associations consistent with longer telomere length indicating slower biological aging. Metabolic signature of DunedinPACE exhibited the most significant associations with diseases and health status, including key aging outcomes such as diabetes, hypertension, functional limitations, and frailty. Notably, all metabolic signatures of EAA showed stronger statistical significance in their associations with renal‐, hepatic‐, and metabolic‐related clinical indicators than with other phenotypic domains, and diseases affecting these systems (gallstones, chronic kidney disease, and hepatitis) were consistently associated with multiple metabolic signatures of EAA. These findings suggest that these signatures may capture metabolic alterations related to physiological dysfunction in systems critical for maintaining organismal homeostasis.

For modifiable lifestyle factors, 15 associations remained statistically significant after FDR correction (out of 22 nominally significant associations) (Figure 4B and Table S14). Metabolic signatures of DunedinPACE and DNAmPhenoAA showed the most consistent patterns, each being significantly associated with four lifestyle factors: current smoking, weekly alcohol consumption, and abnormal BMI (obesity or underweight) were positively associated with both metabolic signatures, whereas high‐level physical activity was negatively associated. The remaining metabolic signatures of EAA showed fewer statistically significant associations: metabolic signature of HorvathAA was positively associated with weekly alcohol consumption and abnormal BMI (obesity and overweight); metabolic signature of HannumAA was positively associated with underweight; and metabolic signature of DNAmTLadjAge was negatively associated with current smoking.

Stratified analyses by sex and age group showed similar association patterns with the primary results, with metabolic signatures of DunedinPACE and DNAmPhenoAA still showing the most significant associations (Tables S15–S18). When reassessing the associations in 778 participants from Phase One and 6217 participants from both Phases, we observed patterns highly consistent with those found in participants from Phase Two (Tables S19–S22). Notably, metabolic signatures of EAA yielded more statistically significant associations than EAA itself, suggesting that metabolite‐informed epigenetic aging indicators may enhance phenotypic relevance.

### 3.5 External Replication

**FIGURE 5:**
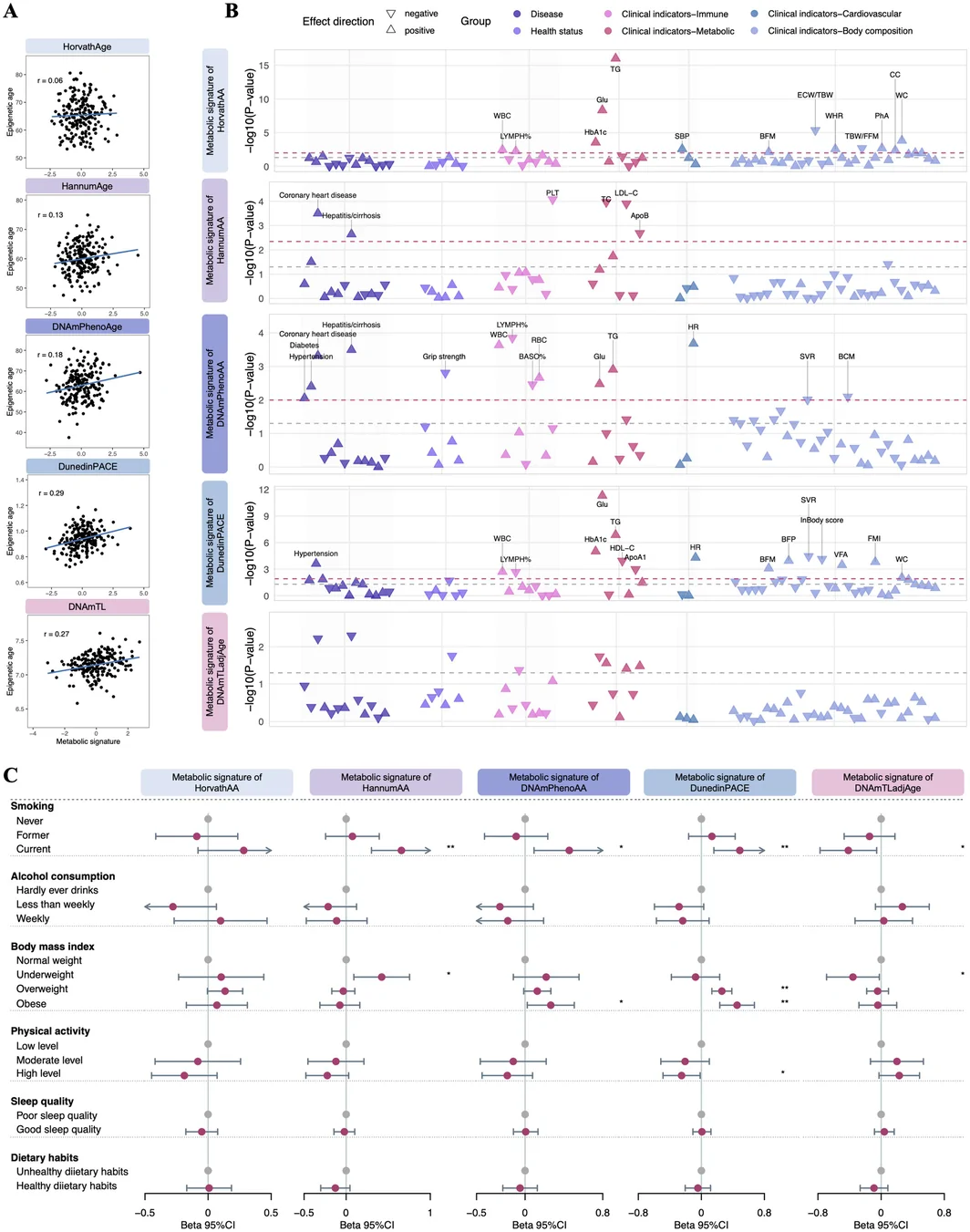
External replication analysis in WCBHC. (A) Scatter plots showing the relationships between each epigenetic aging biomarker and its metabolic signature. Metabolic signatures were standardized to *z*‐scores. Spearman correlation coefficients are indicated. Only metabolic signatures of DNAmPhenoAge, DunedinPACE, and DNAmTL showed significant correlations with their corresponding epigenetic aging biomarkers. (B) Manhattan plot showing associations between metabolic signatures of EAA and aging‐related phenotypes across multiple physiological systems. Upper triangles represent positive effect sizes and lower triangles represent negative effect sizes. The gray dashed line indicates *p* of 0.05 and the red dashed line indicates *p*_FDR_ of 0.05. Phenotypes with *p*_FDR_ < 0.05 are labeled with their names in the plot. Color coding for aging‐related phenotypes and effect direction are shown in the figure legend. (C) Forest plot of associations between modifiable risk factors and metabolic signatures of EAA. Circles represent the point estimates of beta, and error bars represent 95% confidence intervals. Gray represents the reference groups, whereas dark pink represents estimates of each group compared to the reference group. Two asterisks (**) represent *p*_FDR_ < 0.05, and one asterisk (*) represents *p* < 0.05. EAA, epigenetic age acceleration; WCBHC, West China Bone Health Cohort.

In 200 WCBHC participants with both omics data, we replicated seven nominally significant metabolites with concordant directions: three with DunedinPACE (L‐serine, alpha‐lactose, and D‐glucose) and four with DNAmTL (hydroxyphenyllactic acid, phenyllactic acid, fumaric acid, and glutaric acid) (Table S23). Consistent with the primary findings, metabolic signatures of DNAmPhenoAge, DunedinPACE, and DNAmTL were significantly correlated with their corresponding epigenetic aging biomarkers (*r* = 0.18–0.29, all *p* < 0.05, Figure 5A and Table S11), explaining 3.2%–8.6% of the variance, with the metabolic signature of DunedinPACE showing the strongest correlation (*r* = 0.29). Nevertheless, no significant correlations were observed between metabolic signatures of HorvathAge (*r* = 0.06, *p* = 0.40) or HannumAge (*r* = 0.13, *p* = 0.06) and their corresponding epigenetic aging biomarkers in WCBHC.

In 945 WCBHC participants with metabolomic data, we identified 49 statistically significant associations (out of 83 nominally significant associations) between the five metabolic signatures of EAA and aging‐related phenotypes (Figure 5B and Table S24). The metabolic signature of DunedinPACE still exhibited the largest number of significant associations (*n* = 16), followed by the metabolic signature of DNAmPhenoAA (*n* = 14), the metabolic signature of HorvathAA (*n* = 13), and the metabolic signature of HannumAA (*n* = 6), whereas no statistically significant associations were observed for the metabolic signature of DNAmTLadjAge, possibly due to the smaller sample size. The directions and patterns of associations were largely consistent with the primary analysis, though renal‐ and hepatic‐related indicators could not be assessed due to data limitations. For modifiable lifestyle factors, replicated significant associations were observed for smoking (with the metabolic signatures of DunedinPACE and HannumAA) and BMI (with the metabolic signature of DunedinPACE) after FDR correction (Figure 5C and Table S25). In the sensitivity analysis including participants younger than 50 years (total *n* = 1005), the overall patterns, effect directions, and main significant associations remained largely unchanged, suggesting that the observed associations were not materially driven by the exclusion of younger individuals (Tables S26 and S27).

## 4 Discussion

This study characterized the metabolic underpinnings of five established epigenetic aging biomarkers and investigated the clinical relevance and potential determinants of the integrative metabolic signatures in Chinese older adults from two cohorts. Although some metabolites and pathways were shared across epigenetic aging biomarkers, each also exhibited some distinct metabolic correlates. The resulting metabolic signatures of EAA were significantly associated with a broad range of aging‐related phenotypes and modifiable lifestyle factors. Among them, metabolic signatures of DunedinPACE and DNAmPhenoAA exhibited the most extensive associations in both primary and validation cohorts. These findings reveal the metabolic underpinnings of epigenetic aging biomarkers and highlight the potential of metabolomics‐informed epigenetic aging metrics for reflecting physiological decline and lifestyle effects.

Aging is accompanied by systemic metabolic alterations (Khalaf et al. 2025; Jasbi et al. 2023), yet few studies have directly characterized the metabolic correlates of epigenetic aging. One recent study of 4181 German individuals identified specific lipidomic profiles, such as even‐chain ceramides and lysophosphatidylcholines, associated with second‐generation epigenetic clocks; however, it was restricted to lipid metabolism and focused solely on a single generation of clocks (Liu et al. 2023). In this study, we profiled plasma metabolites associated with five established epigenetic aging biomarkers in Chinese older adults, extending previous findings by revealing both shared and specific metabolic profiles. Notably, carbohydrate metabolism emerged as one of the core pathways, with D‐xylose, D‐xylulose, rhamnose, and D‐glucose associated with multiple epigenetic aging biomarkers. These metabolites are involved in energy production, detoxification, and oxidative stress response, which are closely linked to aging‐related adverse outcomes (Khalaf et al. 2025; Ho et al. 2019; Calabrese et al. 2010; Chia et al. 2018). Dysregulation of bile acid metabolism (e.g., ursodeoxycholic acid, cholic acid, glycocholic acid, and glycolithocholic acid) was also prominent, particularly for HorvathAge, HannumAge, DNAmPhenoAge, and DNAmTL, aligning with previous reports implicating bile acids in inflammation, gut barrier integrity, and metabolic regulation during aging (Jin et al. 2024; Perino et al. 2021; Sato et al. 2021). In addition, branched‐chain amino acid (BCAA) metabolic perturbations were observed across epigenetic aging biomarkers, manifesting as either accumulation of BCAA metabolites (e.g., 2‐hydroxy‐3‐methylbutyric acid, 3‐methyl‐2‐oxovaleric acid) or imbalanced BCAA profiles (e.g., L‐valine, L‐isoleucine), indicating mitochondrial dysfunction, insulin resistance, and impaired protein homeostasis (Mann et al. 2021; Grahnemo et al. 2023; Tanase et al. 2024). Glycine and serine metabolism was also implicated across epigenetic aging biomarkers, and both glycine and serine were inversely associated with DNAmPhenoAge and DunedinPACE. This pattern is consistent with prior experimental evidence showing that dietary glycine supplementation modestly extended lifespan in mice and that serine‐related longevity effects have been reported in *Caenorhabditis elegans* (Lionaki et al. 2022; Miller et al. 2019). These findings highlight a potential role of glycine/serine‐related metabolism in the biological aging process. Notably, among all epigenetic aging biomarkers, DunedinPACE exhibited the broadest and most diverse metabolic associations. Moreover, metabolic signature of DunedinPACE showed the most consistent associations across training, internal validation, and external replication cohorts, underscoring its robustness and generalizability as a metabolomics‐informed epigenetic aging indicator. However, this finding warrants further investigation in future studies.

The clinical relevance of the integrative metabolic signatures was supported by consistent associations with a broad range of aging‐related phenotypes in a large independent population and an external validation cohort. Our findings suggest that older adults with accelerated metabolomics‐informed epigenetic aging are more likely to have higher risks of multiple diseases, adverse health states, and unfavorable clinical indicators, consistent with previous studies of epigenetic aging (Kuiper et al. 2023; Faul et al. 2023; Bao et al. 2025; Surachman et al. 2026). Among these associations, we observed particularly strong and consistent patterns with clinical indicators reflecting hepatic, renal, and metabolic function. This may reflect the central role these organ systems play in aging‐related functional decline, as their impairment compromises essential functions such as detoxification, fluid–electrolyte balance, and energy metabolism, thereby contributing to broader physiological decline (Chia et al. 2018; Fang et al. 2020; Hunt et al. 2019; Grant 1991; Ellison and Farrar 2018). Furthermore, these organ systems are tightly linked to circulating metabolites: hepatic metabolism governs metabolite production and clearance, renal function influences elimination kinetics, and metabolic dysregulation alters substrate utilization patterns (Palmer and Jensen 2022; Kalim and Rhee 2017; Rui 2014). Therefore, alterations in metabolite profiles may closely reflect functional decline in these key systems, which may underlie the heightened sensitivity of the metabolic signatures of EAA to clinical indicators from these systems compared to more distal phenotypic outcomes.

Our results identified several modifiable lifestyle factors associated with metabolic signatures of EAA, suggesting the potential value of these metrics for evaluating lifestyle effects, although longitudinal validation is still warranted. Four modifiable lifestyle factors, including smoking status, alcohol consumption, BMI, and physical activity, showed varying degrees of association with different metabolic signatures of EAA, all of which have been previously linked to epigenetic aging (Chervova et al. 2024; Ryan et al. 2020). A previous meta‐analysis of 61 studies reported that BMI was the most consistently associated modifiable factor, showing positive associations with epigenetic aging biomarkers (Ryan et al. 2020). In our study, BMI emerged as the most consistently associated factor across all metabolic signatures of EAA, with both overweight/obesity and underweight associated with accelerated metabolomics‐informed epigenetic aging, highlighting the importance of maintaining a normal weight range in older adults.

Our findings underscore the promise of metabolic signatures for DunedinPACE and DNAmPhenoAA as potential indicators of physiological decline and lifestyle effects. Prior evidence suggests that second‐ and third‐generation epigenetic clocks outperform first‐generation clocks in reflecting health outcomes and lifestyle exposures (Kuiper et al. 2023; Thomas et al. 2024; Whitman et al. 2024; Barr et al. 2025; Yamada 2025). This likely stems from inherent differences in their design: first‐generation clocks were developed to estimate chronological age, whereas second‐generation clocks integrated aging‐related outcomes and third‐generation clocks captured longitudinal change and multi‐system functional decline, providing a more comprehensive reflection of physiological deterioration (Hannum et al. 2013; Horvath 2013; Levine et al. 2018; Belsky et al. 2022). Additionally, DNAmTL, as a proxy for telomere attrition, has shown comparable performance to first‐generation clocks but inferior to second‐generation clocks in reflecting health outcomes (Lu et al. 2019). Our study supported stronger phenotypic relevance of DunedinPACE and DNAmPhenoAA over the other EAA, and further demonstrated the broader associations of their metabolic signatures with both adverse health outcomes and key lifestyle determinants. Interestingly, within the same population of Phase One, metabolic signatures of EAA identified more associations than EAA itself. One explanation lies in the inherent nature of metabolites: as highly dynamic molecules, they can provide integrative gene–environment interaction readout, allowing them to more closely reflect phenotypically relevant variation (Huang, Chen, et al. 2025).

Several limitations should be acknowledged. First, the cross‐sectional design precludes causal inference. The observed associations between epigenetic aging biomarkers and metabolites may be bidirectional. Furthermore, we were unable to evaluate whether the metabolic signatures predict subsequent health outcomes due to the lack of longitudinal data. Second, although our analyses identified epigenetic aging‐associated metabolites and metabolite‐based signatures at the population level, future experimental studies are needed to determine whether key metabolites regulate cellular DNA methylation levels and whether they influence clock‐related CpG methylation patterns by modulating methyltransferase or demethylase activity. Third, the metabolomics platform was based on a targeted panel and may therefore have missed broader metabolic perturbations, which could be captured in future studies using untargeted metabolomic profiling. Fourth, because metabolomic data capture only one layer of biological variation related to epigenetic aging biomarkers, these metabolic signatures were not to totally replace the epigenetic aging biomarkers themselves. Moreover, potential nonlinear associations between metabolites and epigenetic aging biomarkers may not have been fully captured by elastic net regression. Fifth, our focus on older adults may limit the generalizability of the findings to younger populations. Further validation is warranted when extending these findings to younger adults and across the entire adult lifespan. Finally, although most aging‐related phenotypes were assessed in the external cohort, some indicators, such as hepatic‐ and renal‐related clinical indicators, were excluded because they were unavailable or largely missing, warranting future validation.

## 5 Conclusion

To conclude, our work identified a set of metabolites associated with epigenetic aging biomarkers, and these integrated metabolic signatures, especially metabolic signatures of DunedinPACE and DNAmPhenoAA, showed broad associations with adverse health outcomes and key lifestyle determinants. Our findings highlight the potential of metabolomics‐informed epigenetic aging metrics to serve as indicators for evaluating aging‐related physiological decline and lifestyle‐based interventions to promote healthy aging.

## Author Contributions

Conceptualization: B.Z., J.L. (Jiayuan Li), X.J., J.L. (Jiaqiang Liao), T.Z.; data curation: X.Z. (Xunying Zhao), T.M., M.X., X.D., Y.Z., X.C.; formal analysis: X.Z. (Xunying Zhao), T.M., M.X.; funding acquisition: B.Z., J.L. (Jiayuan Li), X.J.; investigation: X.Z. (Xunying Zhao), T.M., M.X., X.D., Y.Z., X.C., Y.Q, B.L., Q.Z., X.W., H.Y, X.Z. (Xingyu Zhang), K.J., L.H.; methodology: X.Z. (Xunying Zhao), T.M., M.X.; project administration: X.C., B.L., Q.Z., H.Y., L.H.; supervision: B.Z., J.L. (Jiayuan Li), X.J., T.Z., J.L. (Jiaqiang Liao), M.F., L.L.; writing – original draft: X.Z. (Xunying Zhao); writing – review and editing: J.L. (Jiayuan Li), X.J.

## Funding

This work was supported by the National Key R&D Program of China (2023YFC3606300), the Science Fund for Creative Research Groups of Science and Technology Bureau of Sichuan Province (2024NSFTD0030), and Sichuan Science and Technology Program (2024NSFSC0050).

## Ethics Statement

The WCHAC and WCBHC were approved by the Ethics Committee of West China Fourth Hospital of Sichuan University (Approval No. for WCHAC: HXSY‐EC‐2022034 and HXSY‐EC‐2025057, Approval No. for WCBHC: HXSY‐EC‐2023029 and HXSY‐EC‐2025059).

## Consent

All participants provided written informed consent.

## Conflicts of Interest

The authors declare no conflicts of interest.

## Supporting information


**Figure S1:** The flowchart diagram of participant selection.
**Figure S2:** Inter‐correlation between metabolic signatures of epigenetic aging biomarkers and epigenetic age acceleration. Inter‐correlation between metabolic signatures of epigenetic aging biomarkers (A) and metabolic signatures of epigenetic age acceleration (B) in the training set. Inter‐correlation between metabolic signatures of epigenetic aging biomarkers (C) and metabolic signatures of epigenetic age acceleration (D) in the testing set. Inter‐correlation between metabolic signatures of epigenetic aging biomarkers (E) and metabolic signatures of epigenetic age acceleration (F) in WCBHC. Asterisks (**) indicate *p*_FDR_ < 0.05.

**Table S1:** CpG site matching rates relative to the original clock designs.
**Table S2:** Details of plasma metabolites.
**Table S3:** Details of aging‐related phenotypes.
**Table S4:** Characteristics of the participants in Phase One from WCHAC.
**Table S5:** Associations of EAA with aging‐related phenotypes across multiple physiological systems.
**Table S6:** Associations of modifiable risk factors with each EAA.
**Table S7:** Spearman correlations between epigenetic aging biomarkers and metabolites.
**Table S8:** Metabolome‐wide associations of epigenetic aging biomarkers.
**Table S9:** Metabolite set enrichment analysis showing the enriched pathways from suggestively significant metabolites in metabolome‐wide associations analysis.
**Table S10:** Non‐zero coefficient metabolites selected by elastic net regression for each epigenetic aging biomarker.
**Table S11:** Spearman correlations between metabolic signatures and the corresponding epigenetic aging biomarkers.
**Table S12:** Characteristics of the participants from WCHAC and WCBHC.
**Table S13:** Associations of metabolic signatures of EAA with aging‐related phenotypes across multiple physiological systems in 5439 participants of Phase Two from WCHAC.
**Table S14:** Associations of modifiable risk factors with each metabolic signature of EAA in 5439 participants of Phase Two from WCHAC.
**Table S15:** Associations of metabolic signatures of EAA with aging‐related phenotypes across multiple physiological systems in 5439 participants of Phase Two from WCHAC by sex.
**Table S16:** Associations of modifiable risk factors with each metabolic signature of EAA in 5439 participants of Phase Two from WCHAC by sex.
**Table S17:** Associations of metabolic signatures of EAA with aging‐related phenotypes across multiple physiological systems in 5439 participants of Phase Two from WCHAC by age groups.
**Table S18:** Associations of modifiable risk factors with each metabolic signature of EAA in 5439 participants of Phase Two from WCHAC by age groups.
**Table S19:** Associations of metabolic signatures of EAA with aging‐related phenotypes across multiple physiological systems in 778 participants of Phase One from WCHAC.
**Table S20:** Associations of modifiable risk factors with each metabolic signature of EAA in 778 participants of Phase One from WCHAC.
**Table S21:** Associations of metabolic signatures of EAA with aging‐related phenotypes across multiple physiological systems in 6217 participants of both Phase One and Phase Two from WCHAC.
**Table S22:** Associations of modifiable risk factors with each metabolic signature of EAA in 6217 participants of both Phase One and Phase Two from WCHAC.
**Table S23:** Metabolome‐wide associations of epigenetic aging biomarkers in 200 participants of WCBHC.
**Table S24:** Associations of metabolic signatures of EAA with aging‐related phenotypes across multiple physiological systems in 945 participants of WCBHC.
**Table S25:** Associations of modifiable risk factors with each metabolic signature of EAA in 945 participants of WCBHC.
**Table S26:** Associations of metabolic signatures of EAA with aging‐related phenotypes across multiple physiological systems in 1005 participants (including participants under the age of 50) of WCBHC.
**Table S27:** Associations of modifiable risk factors with each metabolic signature of EAA in 1005 participants (including participants under the age of 50) of WCBHC.

**Appendix S1:** STROBE Statement—checklist of items that should be included in reports of observational studies.

**Appendix S2:** Method.

## Data Availability

The datasets generated and/or analyzed during the current study are not publicly available due to participant privacy but are available from the corresponding author on reasonable request. This study does not generate any original code. The core code used in this study is available from the corresponding author upon reasonable request.
