# Supplementary material for: Metabolic Profiling of Epigenetic Aging and Its Associations With Aging‐Related Phenotypes and Modifiable Lifestyle Factors
Source: Aging Cell. 2026 Aug 23;25(9):e70657. doi: 10.1111/acel.70657 (PMC13501003; doi:10.1111/acel.70657)
Supplement: Supplementary file 1 [file ACEL-25-e70657-s002.docx]

**Supplementary Figure 1. The flowchart diagram of participant selection.**

**Supplementary Figure 2. Inter-correlation between metabolic signatures of epigenetic aging biomarkers and epigenetic age acceleration.**

**Supplementary Figure 1. The flowchart diagram of participant selection. (A)** Primary analyses were conducted in WCHAC in two phases: Phase One identified metabolic signatures associated with epigenetic aging biomarkers using 778 participants with both methylation and metabolomic data; Phase Two evaluated clinical relevance and determinants of these signatures in 5,439 participants with metabolomic data only. **(B)** External validation was conducted in WCBHC. WCHAC, West China Health and Aging Cohort; WCBHC, West China Bone Health Cohort; EAA, epigenetic age acceleration.

**Supplementary Figure 2. Inter-correlation between metabolic signatures of epigenetic aging biomarkers and epigenetic age acceleration.** Inter-correlation between metabolic signatures of epigenetic aging biomarkers **(A)** and metabolic signatures of epigenetic age acceleration **(B)** in the training set. Inter-correlation between metabolic signatures of epigenetic aging biomarkers **(C)** and metabolic signatures of epigenetic age acceleration **(D)** in the testing set. Inter-correlation between metabolic signatures of epigenetic aging biomarkers **(E)** and metabolic signatures of epigenetic age acceleration **(F)** in WCBHC. Asterisks (**) indicate *P*_FDR_ < 0.05.
