# Supplementary material for: Metabolic Profiling of Epigenetic Aging and Its Associations With Aging‐Related Phenotypes and Modifiable Lifestyle Factors
Source: Aging Cell. 2026 Aug 23;25(9):e70657. doi: 10.1111/acel.70657 (PMC13501003; doi:10.1111/acel.70657)
Supplement: Supplementary file 2 [file ACEL-25-e70657-s003.docx]

**Measurement of DNA methylation**

Genomic DNA was extracted from peripheral blood samples using the MagPure Blood DNA KF Kit (Magen, China). Epigenome-wide DNA methylation profiling was conducted using the Infinium Methylation Screening Array (Illumina, CA, USA), which contains ~270,000 loci across the genome^1^. Raw IDAT files were processed using the ChAMP R package. *β*-values were calculated from probe-specific fluorescent signal intensities according to the Infinium probe-specific algorithms, with values ranging from 0, indicating no methylation, to 1, indicating complete methylation. For quality control, we excluded CpG probes with: (i) detection *P*-value >0.01; (ii) bead count <3 in over 5% of samples; (iii) non-CpG targeting; (iv) minor allele frequency >5% in East Asian populations; (v) cross-hybridization potential; (vi) sex chromosome localization. We excluded samples with: (i) detection *P*-value >0.01 in over 10% of probes; (ii) sex mismatch. A total of 778 participants and 236,685 CpG probes remained. The filtered methylation data were normalized using the BMIQ method to correct probe design bias, followed by batch-effect correction and adjustment for cellular heterogeneity using the ChAMP R package. The final *β*-values were used for downstream analyses and calculation of epigenetic aging biomarkers.

**Measurement of Metabolomics**

**Sample preparation** Plasma samples (−80°C) were thawed at 4°C, then aliquots (20 μL) were mixed with 120 μL isotope internal standard, adequately vortexed and centrifuged (3500 rcf, 20 min). From the resulting supernatant, 30 μL was reacted with 20 μL derivatization reagent and 20 μL 1-Ethyl-3-(3-dimethylaminopropyl)carbodiimide (30°C, 1200 rpm, 60 min). After adding 330 μL 50% methanol and centrifugation (3500 rcf, 4°C, 30 min), 140 μL supernatant was mixed with 10 μL isotope internal standard before ultra performance liquid chromatography-tandem mass spectrometry (UPLC-MS/MS) analysis.

**LC-MS/MS Analysis** The plasma metabolomics profiling was performed using UPLC-MS/MS (TQ-S, Waters, USA) with a high-sensitivity detector for efficient separation and precise detection of derivatized metabolites. Chromatographic separation was performed on a Waters BEH C_18_ column (100 mm × 2.1 mm, 1.7 µm) maintained at 40°C with an injection volume of 5 μL. The mobile phase consisted of solvent A (0.1% formic acid in water) and solvent B (acetonitrile:isopropanol, 7:3, v/v). Data acquisition was performed using Multiple Reaction Monitoring (MRM) mode, simultaneously capturing both positive and negative ionization data. The electrospray ionization (ESI) source parameters were configured as follows: capillary voltage of 3.0 kV (ESI+) and 2.0 kV (ESI−), source temperature of 150°C, desolvation temperature of 550°C, and desolvation gas flow of 1000 L/hr.

Standard stock solutions containing over 300 metabolites were prepared from reference standards. Working standards were diluted to appropriate concentrations and derivatized alongside samples to ensure identical processing conditions. Calibration curves (minimum 5 points) were established for absolute quantification using isotope-labeled internal standards, with complete standard sets analyzed every 64 samples. Quality control (QC) samples were prepared by pooling plasma samples and were processed and analyzed alongside test samples, with one QC sample inserted after every 15 test samples to monitor system stability.

**Data Processing** A total of 221 metabolites were detected in the samples based on the method detection limit. Raw MS data were converted to TXT files using ProteoWizard MSConvert and then imported into TMBQ software for data preprocessing. The workflow included raw data preprocessing, smoothing and denoising, peak identification, alignment, standard curve fitting, and concentration calculation. Peak area ratios of metabolites and internal standards were plotted against the concentrations of the metabolites to obtain calibration curves. The linearity of response was determined by linear regression modeling according to a series of standards at different concentrations in the solvent. Metabolite concentrations below the limit of detection (LOD) were imputed using a left-censored log-normal distribution approach.

**Definition of aging-related phenotypes**

A broad spectrum of aging-related phenotypes was assessed, encompassing self-reported diseases, objective and subjective health status, and clinical indicators reflecting multiple organ systems and physiological functions (**Supplementary Table 3**). Variables with missing data exceeding 20% were excluded from analyses, while variables with less than 20% missing data were imputed using multivariate chained equations.

Self-reported diseases included common chronic conditions, such as metabolic (e.g., diabetes, hyperlipidemia), cardiovascular (e.g., hypertension, coronary heart disease, stroke), respiratory (e.g., chronic bronchitis, asthma, tuberculosis), digestive (e.g., viral hepatitis, steatohepatitis, digestive tract ulcers, gastric polyps, colorectal polyps, chronic atrophic gastritis, gallstones, cholecystitis), genitourinary (e.g., chronic kidney disease), and musculoskeletal diseases (e.g., osteoarthritis, rheumatoid arthritis).

Objective and subjective health status assessments included measures of functional limitations, cognitive impairment, frailty, hearing impairment, anxiety and depression symptoms, grip strength, walking speed, and self-rated health scores. Functional limitations were defined as the inability to independently perform any activity of daily living (ADL) or instrumental activity of daily living (IADL). ADLs included dressing, bathing, feeding, transferring, toileting, and maintaining continence; IADLs included doing housework, cooking, shopping, managing finances, taking medication, and using the telephone^2,3^. Cognitive impairment was defined as a score below education-specific cut-off points on the Chinese version of Mini-Mental State Examination (C-MMSE): <17 for illiterate individuals, <20 for those with primary education, and <24 for those with junior high or higher education^4,5^. Frailty was defined as meeting ≥3 of 5 criteria on the Fried Frailty Phenotype assessment: unintentional weight loss, low grip strength, self-reported exhaustion, slow walking speed, and low physical activity^6^. Hearing impairment was defined as a score ≥8 on the Hearing Handicap Inventory for the Elderly-Screening Version (HHIE-S)^7^. Anxiety was defined as a score ≥10 on the 7-item Generalized Anxiety Disorder Questionnaire (GAD-7)^8^. Depression was defined as a score ≥5 on the 15-item Geriatric Depression Scale (GDS-15)^9,10^. Grip strength was assessed twice using an electronic dynamometer (CAMRY EH101, China), with the maximum value used for analysis. Walking speed was measured as the average of two 6-meter walks at usual pace. Self-rated health was assessed on a 0 to 100 scale, with higher scores indicating better perceived health.

Clinical indicators encompassed commonly used hematological and physical examination indicators from multiple physiological systems, including immune-, metabolic-, renal-, hepatic-, cardiovascular-, and endocrine-related indicators, as well as body composition indicators. Anticoagulated and non-anticoagulated fasting blood samples were collected by trained nurses. Blood biochemical indicators were assessed through standardized laboratory procedures, with estimated glomerular filtration rate (eGFR) calculated using the CKD-EPI (Chronic Kidney Disease Epidemiology Collaboration) equation. Blood pressure was assessed twice using an electronic sphygmomanometer (Omron HBP-1300, Omron, China) after participants rested in a seated position for 5 minutes, with the average of measurements recorded. Liver stiffness and hepatic steatosis were assessed by trained technicians using transient elastography (FibroScan® PRO, Echosens, China). Carotid ultrasonography was performed by trained sonographers using a Hitachi ultrasound system (HV VISION Preirus, Hitachi, Japan) to measure right and left carotid intima-media thickness. Body composition indicators were measured using a bioelectrical impedance analyzer (InBody 770, InBody, Korea) operated by trained researchers. All clinical indicators were log-transformed to reduce the skewed distribution of the data.

**Definition of Covariates and modifiable risk factors**

Socio-demographic factors included chronological age (continuous), sex (female and male), and educational attainment (illiterate, primary, and junior high school or higher). Major occupation was classified as technical and managerial personnel, service and sales personnel, or manual workers. Anthropometric measurements included body mass index (BMI, kg/m²), with participants categorized according to Chinese-specific criteria as underweight (<18.5 kg/m²), normal weight (≥18.5 to <24 kg/m²), overweight (≥24 to <28 kg/m²), and obese (≥28 kg/m²)^11^. Lifestyle factors encompassed smoking status (never, former, and current), alcohol consumption (hardly ever, less than weekly, and weekly), physical activity levels, sleep quality, and dietary habits. Physical activity was assessed using the Global Physical Activity Questionnaire version 2 (GPAQ-2)^12^. Participants were classified into low (<600 MET-minutes/week), moderate (≥600 to <1200 MET-minutes/week), and high (≥1200 MET-minutes/week) physical activity groups^13^. Sleep quality was evaluated using the Pittsburgh Sleep Quality Index (PSQI), with good sleep quality defined as PSQI score ≤5^14^. Dietary habits over the preceding year were assessed via a qualitative food frequency questionnaire. A healthy dietary pattern was defined as meeting at least four of the following five criteria: consuming fresh vegetables ≥7 days/week, fresh fruits ≥7 days/week, red meat 1-6 days/week, legumes ≥4 days/week, and fish ≥1 day/week^15^. Medication history included regular use of diabetes and cardiovascular disease (CVD) medications in the past six months (yes or no).

**Reference**

1.	Goldberg, D. C. *et al.* Scalable screening of ternary-code DNA methylation dynamics associated with human traits. *Cell Genom* **5**, 100929 (2025).

2.	Lawton, M. P. & Brody, E. M. Assessment of older people: self-maintaining and instrumental activities of daily living. *Gerontologist* **9**, 179–186 (1969).

3.	Katz, S., Ford, A. B., Moskowitz, R. W., Jackson, B. A. & Jaffe, M. W. STUDIES OF ILLNESS IN THE AGED. THE INDEX OF ADL: A STANDARDIZED MEASURE OF BIOLOGICAL AND PSYCHOSOCIAL FUNCTION. *JAMA* **185**, 914–919 (1963).

4.	Katzman, R. *et al.* A Chinese version of the Mini-Mental State Examination; impact of illiteracy in a Shanghai dementia survey. *J Clin Epidemiol* **41**, 971–978 (1988).

5.	Li, H., Jia, J. & Yang, Z. Mini-Mental State Examination in Elderly Chinese: A Population-Based Normative Study. *J Alzheimers Dis* **53**, 487–496 (2016).

6.	Fried, L. P. *et al.* Frailty in older adults: evidence for a phenotype. *J Gerontol A Biol Sci Med Sci* **56**, M146-156 (2001).

7.	Ventry, I. M. & Weinstein, B. E. The hearing handicap inventory for the elderly: a new tool. *Ear Hear* **3**, 128–134 (1982).

8.	Spitzer, R. L., Kroenke, K., Williams, J. B. W. & Löwe, B. A brief measure for assessing generalized anxiety disorder: the GAD-7. *Arch Intern Med* **166**, 1092–1097 (2006).

9.	Yesavage, J. A. *et al.* Development and validation of a geriatric depression screening scale: a preliminary report. *J Psychiatr Res* **17**, 37–49 (1982).

10.	Almeida, O. P. & Almeida, S. A. Short versions of the geriatric depression scale: a study of their validity for the diagnosis of a major depressive episode according to ICD-10 and DSM-IV. *Int J Geriatr Psychiatry* **14**, 858–865 (1999).

11.	Zhou, B.-F. & Cooperative Meta-Analysis Group of the Working Group on Obesity in China. Predictive values of body mass index and waist circumference for risk factors of certain related diseases in Chinese adults--study on optimal cut-off points of body mass index and waist circumference in Chinese adults. *Biomed Environ Sci* **15**, 83–96 (2002).

12.	Armstrong, T. & Bull, F. Development of the World Health Organization Global Physical Activity Questionnaire (GPAQ). *J Public Health* **14**, 66–70 (2006).

13.	WHO guidelines on physical activity and sedentary behaviour. https://www.who.int/publications/i/item/9789240015128.

14.	Buysse, D. J., Reynolds, C. F., Monk, T. H., Berman, S. R. & Kupfer, D. J. The Pittsburgh Sleep Quality Index: a new instrument for psychiatric practice and research. *Psychiatry Res* **28**, 193–213 (1989).

15.	Zhu, N. *et al.* Adherence to a healthy lifestyle and all-cause and cause-specific mortality in Chinese adults: a 10-year prospective study of 0.5 million people. *Int J Behav Nutr Phys Act* **16**, 98 (2019).
